# Adverse Childhood Experiences and Adult Household Firearm Ownership

**DOI:** 10.1001/jamanetworkopen.2024.28027

**Published:** 2024-08-15

**Authors:** Alexander Testa, Karyn Fu, Dylan B. Jackson, Daniel C. Semenza, Sandra McKay

**Affiliations:** 1 Department of Management, Policy and Community Health, School of Public Health, University of Texas Health Science Center at Houston, Houston; 2 Rice University, Houston, Texas; 3 Johns Hopkins Bloomberg School of Public Health, Baltimore, Maryland; 4 Department of Sociology, Anthropology, and Criminal Justice, Rutgers University, Camden, New Jersey; 5 Department of Urban-Global Public Health, School of Public Health, Rutgers University, Piscataway, New Jersey; 6 New Jersey Gun Violence Research Center, Rutgers University, Piscataway; 7 Department of Pediatrics, McGovern Medical School, University of Texas Health Science Center at Houston, Houston

## Abstract

This survey study examines exposure to abuse, neglect, and household dysfunction during childhood and likelihood of gun ownership in adulthood.

## Introduction

Adverse childhood experiences (ACEs; defined as abuse, neglect, and household dysfunction before age 18 years) affect behavior and health over the lifespan.^1^ Populations with ACE exposure are vulnerable to greater firearm exposure in childhood and adolescence,^2^ yet the association of ACEs with adult firearm ownership is not well understood, necessitating further research. This survey study used a statewide sample to assess the association between ACEs and household firearm ownership among US adults.

## Methods

This study used cross-sectional survey data from the 2022 Nevada Behavioral Risk Factor Surveillance System (BRFSS), a random-digit-dialing landline and cellular telephone survey on health and health-related behaviors administered in English and Spanish.^3^ Nevada is the only state in the BRFSS that includes questions from the ACEs and Firearms optional state modules. Nevada BRFSS reported a 40.2% response rate in 2022. In accordance with the Common Rule, this study was exempt from ethics review and informed consent requirement because it used publicly available BRFSS data. We followed the AAPOR reporting guideline.

Respondents were asked yes-or-no retrospective questions on ACEs (eTable 1 in Supplement 1), which were summed and categorized into 0, 1, 2 to 3, or 4 or more ACEs.^4^ Respondents also were asked to answer yes or no to the question, “Are any firearms now kept in or around your home?” (eTable 2 in Supplement 1). We used a survey-weighted multivariable logistic regression in Stata 17 (StataCorp LLC) to test the association between cumulative ACE exposure and household firearm ownership, adjusting for covariates described in eTable 3 in Supplement 1. Self-reported race and ethnicity were analyzed in the study because of their association with ACEs and firearm ownership.

Statistical significance was specified at 2-tailed *P* < .05. Data analysis was performed between March and June 2024.

## Results

Among the 1709 adults (814 males [50.9%], 895 females [49.1%]; mean [SD] age, 48.5 [16.7] years) in the sample, 766 (42.4%) reported household firearm ownership (Table 1). Household firearm ownership was lowest among respondents with 0 ACEs (160 [30.8%]) and highest among those with 4 or more ACEs (211 [49.8%]). Multivariable logistic regression demonstrated an association between having 2 to 3 ACEs (adjusted odds ratio [AOR], 1.89; 95% CI, 1.20-2.98; *P* = .006) or 4 or more ACEs (AOR, 2.12; 95% CI, 1.34-3.33; *P* = .001) and household firearm ownership, compared with 0 ACEs. Among individual ACE items, household illegal drug use (AOR, 1.57; 95% CI, 1.05-2.35; *P* = .03) and verbal abuse (AOR, 1.54; 95% CI, 1.12-2.12; *P* = .008) were associated with household firearm ownership (Table 2). Supplemental analyses found that the association of 4 or more ACEs and household firearm ownership was similar among females (AOR, 2.12; 95% CI, 1.10-4.08; *P* = .03) and males (AOR, 1.99; 95% CI, 1.07- 3.70; *P* = .03).

**Table 1.** Summary Statistics of the Analytic Sample From the Nevada Behavioral Risk Factor Surveillance System, 2022^a^

Characteristic	Respondents, No. (%)					*P* value^b^
	Full sample (n = 1709)	0 ACE (n = 409)	1 ACE (n = 394)	2-3 ACEs (n = 442)	≥4 ACEs (n = 464)	
Household firearm ownership						
No	943 (57.6)	249 (69.2)	211 (59.9)	230 (54.9)	253 (50.2)	.001
Yes	766 (42.4)	160 (30.8)	183 (40.1)	212 (45.1)	211 (49.8)	
No. of ACEs						
0	409 (22.2)	NA	NA	NA	NA	NA
1	394 (23.0)	NA	NA	NA	NA	
2-3	442 (25.2)	NA	NA	NA	NA	
≥4	464 (29.6)	NA	NA	NA	NA	
Age, mean (SD), y	48.5 (16.7)	51.5 (17.9)	52.6 (16.3)	49.0 (16.8)	42.5 (14.2)	NA
Sex						
Female	895 (49.1)	212 (50.5)	188 (43.4)	224 (45.3)	271 (55.6)	.06
Male	814 (50.9)	197 (49.5)	206 (56.6)	218 (54.7)	193 (44.4)	
Race and ethnicity^c^						
Hispanic	261 (26.0)	65 (30.2)	59 (27.0)	67 (24.9)	70 (23.1)	.93
Non-Hispanic Black	93 (10.2)	17 (8.0)	21 (10.2)	26 (11.0)	29 (11.1)	
Non-Hispanic White	1217 (47.7)	299 (45.7)	282 (45.2)	314 (48.7)	322 (50.4)	
Non-Hispanic Other^d^	138 (16.1)	28 (16.2)	32 (17.6)	35 (15.4)	43 (15.3)	
Marital status						
Married	785 (49.6)	206 (54.8)	192 (50.0)	208 (49.7)	179 (45.3)	.06
Divorced or separated	334 (15.9)	66 (12.5)	78 (18.7)	81 (14.1)	109 (17.7)	
Widowed	214 (7.6)	71 (10.7)	55 (8.9)	52 (8.1)	36 (3.9)	
Never married	277 (20.5)	46 (15.4)	52 (18.4)	75 (22.2)	104 (24.5)	
Member of an unmarried couple	99 (6.4)	20 (6.7)	17 (3.9)	26 (5.8)	36 (8.6)	
Educational attainment						
<High school	113 (13.1)	28 (16.5)	27 (13.5)	30 (13.9)	28 (9.4)	.006
High school graduate	402 (26.7)	89 (22.6)	80 (24.9)	97 (25.1)	136 (32.5)	
Some college	505 (34.2)	100 (27.1)	118 (34.9)	126 (32.7)	161 (40.3)	
College graduate	689 (26.0)	192 (33.7)	169 (26.7)	189 (28.3)	139 (17.8)	
Child in home						
No	1243 (61.4)	298 (59.3)	308 (68.1)	324 (61.8)	313 (57.4)	.18
Yes	466 (38.6)	111 (40.7)	86 (31.9)	118 (38.2)	151 (42.6)	
Veteran status						
No	1463 (87.6)	346 (89.0)	328 (83.5)	379 (87.9)	410 (89.6)	.21
Yes	246 (12.4)	63 (11.0)	66 (16.5)	63 (12.1)	54 (10.4)	
Household income, US$						
<25 000	243 (14.8)	49 (12.7)	60 (18.5)	57 (11.0)	77 (16.6)	.23
25 000-49 999	459 (27.8)	119 (33.6)	71 (19.5)	116 (28.9)	153 (29.0)	
50 000-74 999	292 (16.4)	64 (15.1)	68 (15.3)	80 (20.1)	80 (15.0)	
75 000-99 999	246 (14.5)	51 (12.2)	70 (18.0)	67 (15.6)	58 (12.6)	
100 000-149 999	259 (13.7)	62 (11.5)	70 (15.1)	65 (13.0)	62 (14.7)	
≥150 000	210 (12.9)	64 (14.9)	55 (13.6)	57 (11.4)	34 (12.1)	

Abbreviations: ACE, adverse childhood experience; NA, not applicable.

^a^ The table presents weighted percentages and unweighted frequencies.

^b^ *P* value represents the results from χ^2^ tests.

^c^ Race and ethnicity were self-reported by survey respondents.

^d^ Non-Hispanic Other category included American Indian or Alaskan Native, Asian, or Pacific Islander.

**Table 2.** Weighted Multiple Logistic Regression of Association Between Household Firearm Ownership and Adverse Childhood Experiences

Characteristic	AOR (95% CI)	*P* value
No. of ACEs^a^^,^^b^		
1	1.38 (0.87-2.20)	.17
2-3	1.89 (1.20-2.98)	.006
≥4	2.12 (1.34-3.33)	.001
Type of ACE^a^^,^^c^		
Household mental illness	1.14 (0.77-1.70)	.51
Household alcoholism	1.27 (0.91-1.77)	.17
Household illegal drug use	1.57 (1.05-2.35)	.03
Household incarceration	1.06 (0.64-1.77)	.82
Parents divorced or separated	1.35 (0.99-1.86)	.06
Household domestic violence	1.27 (0.89-1.82)	.19
Physical abuse	1.67 (0.70-3.98)	.25
Verbal abuse	1.54 (1.12-2.12)	.008
Sexual abuse	1.06 (0.71-1.57)	.78

Abbreviations: ACEs, adverse childhood experiences; AOR, adjusted odds ratio.

^a^ All models include control variables for age, sex, race and ethnicity, marital status, educational attainment, child in the home, veteran status, and household income.

^b^ Reference category for number of ACEs is 0 ACEs.

^c^ Each row under Type of ACE represents a separate multiple logistic regression analysis.

## Discussion

To our knowledge, this study was the first to evaluate the association between ACEs and household firearm ownership in adulthood. Consistent with prior research on ACE exposure and presence of a firearm in the household during childhood,^2^ this study found that cumulative ACE exposure was associated with higher odds of household firearm ownership in adulthood. The relationship may be due to a heightened sense of vulnerability to physical violence and greater perceived threats to personal safety associated with a traumatic childhood, which lead individuals to seek self-protection. Furthermore, exposure to violence and instability in childhood may normalize the presence and use of weapons, making firearm ownership a more acceptable and familiar choice later in life.^5^ Still, having a firearm in the home is a known risk factor for suicide, homicide, and unintentional injury.^6^

Study limitations include data from only 1 state, potential response bias from self-reported data, cross-sectional data, and lack of data on household firearm ownership during childhood. The findings underscored the need for future data from various states on ACEs and measures of firearm ownership, storage patterns, and attitudes.

## Notes

Supplement 1. **eTable 1.** Definitions and Coding of Adverse Childhood Experience Questions **eTable 2.** Definitions and Coding of Household Firearm Ownership **eTable 3.** Definition and Coding of Control Variables

Supplement 2.
Data Sharing Statement
