# Supplementary material for: Adverse Childhood Experiences and Adult Household Firearm Ownership
Source: JAMA Netw Open. 2024 Aug 15;7(8):e2428027. doi: 10.1001/jamanetworkopen.2024.28027 (PMC11327881; doi:10.1001/jamanetworkopen.2024.28027)
Supplement: Supplement 1. — eTable 1. Definitions and Coding of Adverse Childhood Experience Questions eTable 2. Definitions and Coding of Household Firearm Ownership eTable 3. Definition and Coding of Control Variables [file jamanetwopen-e2428027-s001.pdf]

Supplemental Online Content

Testa A, Fu K, Jackson DB, Semenza DC, McKay S. Adverse childhood experiences and adult household firearm ownership. *JAMA Netw Open*. 2024;7(8):e2428027. doi:10.1001/jamanetworkopen.2024.28027

eTable 1. Definitions and Coding of Adverse Childhood Experience Questions

eTable 2. Definitions and Coding of Household Firearm Ownership

eTable 3. Definition and Coding of Control Variables

This supplemental material has been provided by the authors to give readers additional information about their work.

© 2024 Testa A et al. *JAMA Network Open*

eTable 1. Definitions and Coding of Adverse Childhood Experience Questions

Prologue: I'd like to ask you some questions about events that happened during your childhood. This information will allow us to better understand problems that may occur early in life and may help others in the future. This is a sensitive topic and some people may feel uncomfortable with these questions. At the end of this section, I will give you a phone number for an organization that can provide information and referral for these issues. Please keep in mind that you can ask me to skip any question you do not want to answer. All questions refer to the time period before you were 18 years of age.

ACE Item	Question	Original Coding	Coding for Analysis
Household Mental Illness	Did you live with anyone who was depressed, mentally ill, or suicidal?	1 Yes<br>2 No<br>7 Don't Know/Not Sure<br>9 Refused	0 No<br>1 Yes<br>*Don't Know/Not Sure or Refused recoded as missing
Household Alcoholism	Did you live with anyone who was a problem drinker or alcoholic?	1 Yes<br>2 No<br>7 Don't Know/Not Sure<br>9 Refused	0 No<br>1 Yes<br>*Don't Know/Not Sure or Refused recoded as missing
Household Illegal Drug Use	Did you live with anyone who used illegal street drugs or who abused prescription medications?	1 Yes<br>2 No<br>7 Don't Know/Not Sure<br>9 Refused	0 No<br>1 Yes<br>*Don't Know/Not Sure or Refused recoded as missing
Household Incarceration	Did you live with anyone who served time or was sentenced to serve time in a prison, jail, or other correctional facility?	1 Yes<br>2 No<br>7 Don't Know/Not Sure<br>9 Refused	0 No<br>1 Yes<br>*Don't Know/Not Sure or Refused recoded as missing
Parents Divorced or Separated	Were your parents separated or divorced?	1 Yes<br>2 No<br>7 Don't Know/Not Sure<br>8 Parents not married<br>9 Refused	0 No or not Married<br>1 Yes<br>*Don't Know/Not Sure or Refused recoded as missing
Household Domestic Violence	How often did your parents or adults in your home ever slap, hit, kick, punch or beat each other up? Was it…	1 Never<br>2 Once<br>3 More than once<br>7 Don't Know/Not Sure<br>9 Refused	0 Never<br>1 Once or more<br>*Don't Know/Not Sure or Refused recoded as missing
Physical Abuse	Not including spanking, (before age 18), how often did a parent or adult in your home ever hit, beat, kick, or physically hurt you in any way? Was it…	1 Never<br>2 Once<br>3 More than once<br>7 Don't Know/Not Sure<br>9 Refused	0 Never<br>1 Once or more<br>*Don't Know/Not Sure or Refused recoded as missing
Verbal Abuse	How often did a parent or adult in your home ever swear at you, insult you, or put you down? Was it…	1 Never<br>2 Once<br>3 More than once<br>7 Don't Know/Not Sure<br>9 Refused	0 Never<br>1 Once or more<br>*Don't Know/Not Sure or Refused recoded as missing

© 2024 Testa A et al. *JAMA Network Open*

Sexual Abuse	(1) How often did anyone at least 5 years older than you or an adult, ever touch you sexually? Was it…<br><br>(2) How often did anyone at least 5 years older than you or an adult, try to make you touch them sexually? Was it…<br><br>(3) How often did anyone at least 5 years older than you or an adult, force you to have sex? Was it…	1 Never<br>2 Once<br>3 More than once<br>7 Don't Know/Not Sure<br>9 Refused	0 Never to all 3 questions<br>1 Once or more to any of the 3 questions<br>*Don't Know/Not Sure or Refused recoded as missing

© 2024 Testa A et al. *JAMA Network Open*

eTable 2. Definitions and Coding of Household Firearm Ownership

<table>
<tr><td colspan="4"><u>Prologue:</u> The next questions are about safety and firearms. Some people keep guns for recreational purposes such as hunting or sport shooting. People also keep guns in the home for protection. Please include firearms such as pistols, revolvers, shotguns, and rifles; but not BB guns or guns that cannot fire. Include those kept in a garage, outdoor storage area, or motor vehicle.</td></tr>
<tr><th>ACE Item</th><th>Question</th><th>Original Coding</th><th>Coding for Analysis</th></tr>
<tr><td>Household Firearm Ownership</td><td>Are any firearms now kept in or around your home?</td><td>1 Yes
2 No
7 Don't Know/Not Sure
9 Refused</td><td>0 No
1 Yes
*Don't Know/Not Sure or Refused recoded as missing</td></tr>
</table>

© 2024 Testa A et al. *JAMA Network Open*

eTable 3. Definition and Coding of Control Variables

Variable	Question	Original Coding	Coding for Analysis
Age	What is your age?	Age in years	Age in years
Sex	Are you male or female?	1 Male<br>2 Female	0 Female<br>1 Male
Self-Reported Race/Ethnicity	(1) Which one or more of the following would you say is your race?<br><br>(2) Are you Hispanic, Latino/a, or Spanish origin?<br><br>*Note – Race/ethnicity is classified using two separate questions inquiring about racial background, and separately Hispanic, Latino/a, or Spanish ethnicity.	**(1) Race**<br>10 White<br>20 Black or African American<br>30 American Indian or Alaska Native<br>40 Asian<br>41 Asian Indian<br>42 Chinese<br>43 Filipino<br>44 Japanese<br>45 Korean<br>46 Vietnamese<br>47 Other Asian<br>50 Pacific Islander<br>51 Native Hawaiian<br>52 Guamanian or Chamorro<br>53 Samoan<br>54 Other Pacific Islander<br>88 No choices<br>77 Don't know / Not sure<br>99 Refused<br><br>**(2) Hispanic/Latino/a Ethnicity**<br>Are you…<br>1 Mexican, Mexican American, Chicano/a<br>2 Puerto Rican<br>3 Cuban<br>4 Another Hispanic, Latino/a, or Spanish origin<br>5 No<br>7 Don't know / Not sure<br>9 Refused	0 Hispanic<br>1 non-Hispanic Black<br>2 non-Hispanic White<br>3 non-Hispanic Other Race<br><br>*Don't Know/Not Sure or Refused recoded as missing

© 2024 Testa A et al. *JAMA Network Open*

Variable	Question	Original Coding	Coding for Analysis
Marital Status	Are you…	1 Married<br>2 Divorced<br>3 Widowed<br>4 Separated<br>5 Never married<br>6 A member of an unmarried couple<br>9 Refused	1 Married<br>2 Divorced or Separated<br>3 Widowed<br>4 Never married<br>5 A member of an unmarried couple<br>*Refused recoded as missing
Educational Attainment	What is the highest grade or year of school you completed?	1 Never attended school or only attended kindergarten<br>2 Grades 1 through 8 (Elementary)<br>3 Grades 9 through 11 (Some high school)<br>4 Grade 12 or GED (High school graduate)<br>5 College 1 year to 3 years (Some college or technical school)<br>6 College 4 years or more (College graduate)<br>9 Refused	0 Less than high school<br>1 High school graduate<br>2 Some college<br>3 College graduate<br>*Refused recoded as missing
Child in Home	How many children less than 18 years of age live in your household?	-- Number of children<br>88 None<br>99 Refused	0 No children<br>1 One or more Children<br>*Don't Know/Not Sure or Refused recoded as missing
Military Veteran	Have you ever served on active duty in the United States Armed Forces, either in the regular military or in a National Guard or military reserve unit?	1 Yes<br>2 No<br>7 Don't know / Not sure<br>9 Refused	0 No<br>1 Yes<br>*Don't Know/Not Sure or Refused recoded as missing
Income	Is your annual household income from all sources:	1 Less than $10,000?<br>2 Less than $15,000? ($10,000 to less than $15,000)<br>3 Less than $20,000? ($15,000 to less than $20,000)<br>4 Less than $25,000	1 Less than $25,000<br>2 $25,000 - $49,999<br>3 $50,000 - $74,999<br>4 $75,000 - $99,999<br>5 $100,000 - $149,000<br>6 $150,000 or more

© 2024 Testa A et al. *JAMA Network Open*

Variable	Question	Original Coding	Coding for Analysis
		5 Less than $35,000 If ($25,000 to less than $35,000)<br>6 Less than $50,000 If ($35,000 to less than $50,000)<br>7 Less than $75,000? ($50,000 to less than $75,000)<br>8 Less than $100,000? ($75,000 to less than $100,000)<br>9 Less than $150,000? ($100,000 to less than $150,000)?<br>10 Less than $200,000? ($150,000 to less than $200,000)<br>11 $200,000 or more<br>77 Don't know / Not sure<br>99 Refused	*Don't Know/Not Sure or Refused recoded as missing

© 2024 Testa A et al. *JAMA Network Open*
