# Supplementary material for: Adverse Childhood Experiences and Adult Household Firearm Ownership
Source: JAMA Netw Open. 2024 Aug 15;7(8):e2428027. doi: 10.1001/jamanetworkopen.2024.28027 (PMC11327881; doi:10.1001/jamanetworkopen.2024.28027)
Supplement: Supplement 2. — Data Sharing Statement [file jamanetwopen-e2428027-s002.pdf]

Data Sharing Statement

Testa. Adverse Childhood Experiences and Adult Household Firearm Ownership. *JAMA Netw Open*. Published August 15, 2024. doi:10.1001/jamanetworkopen.2024.28027

Data
Data available: Yes
Data types: Deidentified participant data
How to access data: Data are publicly available at: https://www.cdc.gov/brfss/index.html
When available: With publication

Supporting Documents
Document types: Statistical/analytic code
How to access documents: Code will be emailed upon request: alexander.testa@uth.tmc.edu
When available: With publication

Additional Information
Who can access the data: anyone requesting the data
Types of analyses: for any purpose
Mechanisms of data availability: without investigator support
